# C-reactive protein is a predictor for lower-extremity deep venous thrombosis in patients with primary intracerebral hemorrhage

**DOI:** 10.1186/s40001-024-01842-3

**Published:** 2024-06-06

**Authors:** Gang Wang, Bao-Fang Wu, Wen-Jun Zhao, Wei-Peng Hu, Jia-Yin Wang, Hong-Zhi Gao

**Affiliations:** 1 Department of Neurosurgery, The Second Affiliated Hospital of Fujian Medical University, Quanzhou, China; 2 Department of Neurosurgery, The Second Affiliated Clinical Medical College of Fujian Medical University, Quanzhou, China; 3 Department of Neurosurgery, The Second Hospital & Clinical Medical School, Lanzhou University, Lanzhou, China; 4 Key Lab of Neurology of Gansu Province, Lanzhou, China; 5 Department of Health Management Center, The Second Hospital & Clinical Medical School, Lanzhou University, Lanzhou, China

**Keywords:** C-reactive protein, Deep vein thrombosis, Inflammation, Intracerebral hemorrhage, Dose–response relationship

## Abstract

**Objective:**

Our study aimed to determine whether there exists an association between low-grade systemic inflammation, as measured by serum C-reactive protein (CRP), and the risk of lower-extremity deep venous thrombosis (LEDVT) in patients with primary intracerebral hemorrhage (ICH).

**Methods:**

This observational study was retrospectively conducted on patients with primary ICH who were presented to two tertiary medical centers between January 2021 and August 2022. The primary outcome was detecting LEDVT occurrence within 14 days from the onset of the acute ICH episode. Weighted logistic regression and restricted cubic spline models were employed to estimate the association between CRP and LEDVT following 1:1 propensity score matching (PSM).

**Results:**

Of the 538 patients with primary ICH who met the inclusion criteria, 76 (14.13%) experienced LEDVT. Based on the cut-off levels of CRP measured upon admission from the receiver operating characteristic (ROC) curve, patients with primary ICH were categorized into two groups: (i) CRP < 1.59 mg/L and (ii) CRP ≥ 1.59 mg/L. After 1:1 PSM, the LEDVT events occurred in 24.6% of patients with CRP ≥ 1.59 mg/L and 4.1% of patients with CRP < 1.59 mg/L (*P* < 0.001). ROC curve revealed the area under the ROC curve of 0.717 [95% confidence interval (CI) 0.669–0.761, *P* < 0.001] for CRP to predict LEDVT with a sensitivity of 85.71% and specificity of 56.29%. After adjusting for all confounding variables, the occurrence of LEDVT in ICH patients with higher CRP levels (≥ 1.59 mg/L) was 10.8 times higher compared to those with lower CRP levels (95% CI 4.5–25.8, *P* < 0.001). A nonlinear association was observed between CRP and an increased risk of LEDVT in the fully adjusted model (*P* for overall < 0.001, *P* for nonlinear = 0.001). The subgroup results indicated a consistent positive link between CRP and LEDVT events following primary ICH.

**Conclusions:**

Higher initial CRP levels (CRP as a dichotomized variable) in patients with primary ICH are significantly associated with an increased risk of LEDVT and may help identify high-risk patients with LEDVT. Clinicians should be vigilant to enable early and effective intervention in patients at high risk of LEDVT.

**Supplementary Information:**

The online version contains supplementary material available at 10.1186/s40001-024-01842-3.

## Introduction

Lower extremity deep vein thrombosis (LEDVT) is a common complication following primary intracerebral hemorrhage (ICH). It is a clinically devastating condition, with approximately 10.0% to 49.6% of immobilized post-stroke patients suffering from deep vein thrombosis (DVT) [1–5]. Post-stroke LEDVT can contribute to fatal pulmonary embolism (PE), which is the predominant cause of mortality in stroke patients, accounting for 25%–33% of deaths following acute stroke [6]. LEDVT after ICH affects the initial disease’s transformation and regression, and increases the risk of death.

The principal risk factors for LEDVT include immobilization, surgery, and hypercoagulation disorders [7–9]. Consequently, several unknown factors may be involved in the pathogenesis of LEDVT. Identifying risk factors for LEDVT with predictive or causal relevance can help develop prevention strategies, particularly in a high-risk population such as acute ICH [8, 10]. Risk predictors of LEDVT in patients with ICH, including elderly, severity [modified Rankin Scale score (mRS): 3–5], paralysis, immobilization, infection, and laboratory characteristics, have been previously established in the literature [4, 9, 11].

Serum biomarkers can facilitate the prediction of the onset and progression of LEDVT following ICH and make early decisions on the prevention and therapeutic direction of LEDVT [4, 12, 13]. Elevated white blood cell count values, d-dimer, platelet–lymphocyte ratio, and d-dimer-to-fibrinogen ratio are associated with LEDVT after ICH [4, 12–14]. Of the serum biomarkers reported in the literature to date, inflammatory biomarkers are among the widest and most popular studied indicators of vascular inflammation. Undeniably, inflammation is well established as a crucial factor in the occurrence and progression of DVT, including LEDVT following ICH [4, 13–15]. Our previous study has identified leukocytes upon admission as an independent predictor of risk for LEDVT in elderly patients with primary ICH [4].

C-reactive protein (CRP) is an easily accessible positive acute-phase protein, a highly sensitive, non-specific marker of systemic inflammation, which is elevated following ICH, tissue injury, or inflammation [16]. Serum CRP increases with established risk factors for venous thromboembolism (VTE), such as advancing age, obesity, immobilization, and malignancy [16–19]. The number of studies on the associations between CRP and VTE is limited, and the results are controversial. CRP is independently associated with VTE and may be valuable in identifying and stratifying individuals at risk for VTE events in primary care patients [20]. Elevated circulating CRP was associated with an increased risk of VTE in a prospective study and meta-analysis and agreed with a linear dose–response relationship [7]. However, a review found weak evidence for a causal role of CRP in VTE etiology [21]. The Tromsø study was a large prospective study suggesting no association between serum CRP levels and subsequent risk of VTE [22]. Genetic polymorphisms that increase CRP levels have not been associated with increased VTE risk [23]. Yet, the role of CRP in LEDVT is poorly understood, and few studies have investigated the association between CRP and LEDVT following primary ICH. Conversely, the accuracy of the relationship between CRP and LEDVT may be influenced by baseline data such as different sociodemographic characteristics and disease backgrounds. Therefore, it is essential to utilize propensity score matching (PSM) analysis to address any imbalance in baseline data and effectively demonstrate the connection between CRP and LEDVT.

Our study aimed to assess whether low-grade systemic inflammation, measured by serum CRP, was associated with the risk of LEDVT in two tertiary-center retrospective cohort studies of patients with primary ICH following PSM.

## Materials and methods

### Study population

**Fig. 1:**
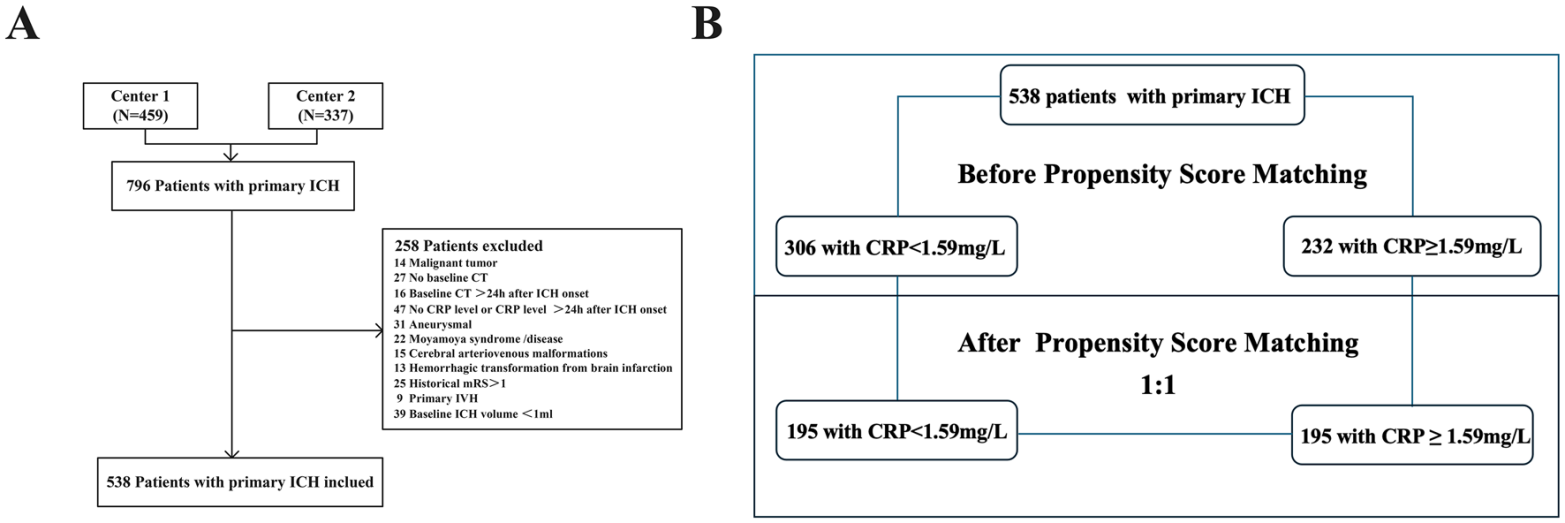
Flowchart of the study. **A** Flowchart of patients’ inclusion and exclusion. **B** Flowchart of propensity-score matching. Centre 1 = The Second Affiliated Hospital of Fujian Medical University Centre 2 = The Second Hospital & Clinical Medical School of Lanzhou University. *CRP* C-reactive protein, *CT* computed tomography, *ICH* intracerebral hemorrhage

This retrospective, observational study was conducted from two tertiary medical centers (The Second Affiliated Hospital of Fujian Medical University and The Second Hospital & Clinical Medical School of Lanzhou University) on consecutive patients presenting with primary ICH between January 2021 and August 2022. The Ethics Committee of The Second Affiliated Hospital of Fujian Medical University and The Second Hospital & Clinical Medical School of Lanzhou University approved the uniform study protocol. In accordance with the national legislation and institutional requirements, patient’s informed consent is not required for retrospective studies. Data for the present study were retrieved from electronic medical records incorporating demographic, clinical, surgical, and follow-up information. The inclusion criteria for the patients observed in the present study were as follows: (a) patients required an index ICH admission computed tomography (CT) scan (for diagnostic confirmation and hematoma localization) within 24 h of the onset of symptoms; (b) follow-up CT scans, CT angiography (CTA) and/or digital subtraction angiography (DSA) were performed with 24 h after baseline CT scan; (c)peripheral blood samples were drawn by venipuncture of a peripheral vein from patients admittance to the unit; (d) LEDVT was verified by Doppler ultrasonography. Exclusion criteria were as follows: (a) malignant tumor; (b) diagnoses with secondary ICH, including intracerebral aneurysm, cerebral arteriovenous malformation, Moyamoya disease, brain tumor, and the hemorrhagic transformation from brain infarction; (c) no CRP level or CRP level > 24 h after ICH onset; (d) primary intraventricular hemorrhage (IVH); (e) historical stroke or historical mRS score > 2; (f) baseline ICH volume < 1 mL; (g) history of DVT or pulmonary embolism (PE); (h) history of thrombophilia. The study design flowchart is depicted in Fig. 1.

### Baseline data collection

Demographic information, clinical history, vital signs, laboratory findings, treatments, complications, and outcomes during hospitalization were extracted from electronic medical records and analyzed for the patients enrolled. Laboratory examinations, including blood routine, biochemical blood indexes, d-dimer, CRP, and coagulation, were performed by venipuncture of peripheral venous blood within one hour of admission. Admission laboratory tests included hemoglobin (Hb), platelet (PLT), prothrombin time (PT), international normalized ratio (INR), activated partial thromboplastin time (aPTT), d-dimer and C-reactive protein (CRP). The CRP (reference range, 0–10 mg/L) was collected from the biochemical blood indexes. The hematoma volume was manually measured by two radiologists with 5 or 6 years of experience in the diagnosis of ICH and calculated using the ABC/2 method as described previously [24]. Deep ICH was defined as selective involvement of the thalamus, basal ganglia, internal capsule, and deep periventricular white matter, whereas lobar ICH originated from the cortex and cortical–subcortical junction [24, 25]. ICH hematoma expansion was defined as hematoma growth with a 33% or 6 mL increase in hematoma on the follow-up CT taken at 24 h [26–28].

### Outcome assessments

The predominant endpoint was the occurrence of LEDVT within 14 days of the acute ICH episode. LEDVT was defined as proximal thrombosis of the iliac or superficial femoral vein, or thrombosis of at least the upper third part of the deep calf veins [29], and was based on lower-extremity ultrasonography showing non-compressibility of a distal or proximal vein [30]. Thrombosis located in the popliteal vein and/or above is regarded as proximal DVT, while those below the popliteal vein are regarded as distal DVT [5, 31, 32].

For patients with signs or symptoms of LEDVT (e.g., swelling, pain), the diagnosis of LEDVT was made by full-color Doppler ultrasound (CDU) waveforms and images from the thigh to the ankle [32]. The following signs confirmed it: directly visible intraluminal thrombus, loss of venous compressibility, dilated veins larger in diameter than the adjacent artery, blunted or absent augmentation flow, and lack of spontaneous flow [30, 32]. CDU is routinely performed weekly for neuro ICU inpatients and once every two weeks for neurosurgery inpatients [4].

The Caprini risk assessment score is employed to enhance the prevention of venous thromboembolism and to determine the required thromboprophylaxis modalities accordingly [33]. Thrombosis was deemed triggered if at least one of the following criteria was present: paralysis of the symptomatic leg, recent trauma (within the past 60 days), bedridden for more than 3 days, and hospitalization within the previous 6 months [29]. If a negative CDU result was reported, a lower-extremity intermittent pneumatic compression was routinely administered to prevent LEDVT. Therapeutic anticoagulants would be initiated for patients with LEDVT to fully resolve symptoms according to institutional protocols. Low-molecular-weight heparin (100 U/kg, Q12H) was prescribed in ICH patients with acute LEDVT after the CDU confirmed the diagnosis and assessment of the risk of rebleeding [4].

### Statistical analysis

For all analyses, a two-sided *P-*value less than 0.05 denoted statistical significance. All statistical analyses were undertaken using SPSS software (version 25.0, IBM SPSS, IBM Corp, USA), MedCalc version 20.0.4 (MedCalc Software, Ostend, Belgium) and R software (version 4.2.1). Quantitative variables are presented as means (± standard deviations, SD) or medians (interquartile ranges, IQR), and categorical variables as counts (percentages). The Kolmogorov–Smirnov (KS) test was performed on each variable to test for normal normality. Continuous variables were compared with the Student’s *t*-test (normal distribution) and nonparametric Mann–Whitney *U* test (skew distribution). Tests for differences between categorical variables employed the *χ*^2^ or Fisher’s exact tests.

To deal with the issue of multicollinearity caused by multiple covariates, we performed the multilinearity diagnosis that involved all variables. We assessed tolerance and variance inflation factors (VIFs) as diagnostic tools to identify multicollinearity. If a VIF is greater than five or the tolerance is less than 0.2, it suggests the presence of multiple collinearities [34].

A univariate analysis followed by a multivariate logistic regression was conducted to assess the risk factors for LEDVT. All variables were regarded as potential risk factors when *P* < 0.10 in the univariable analysis and were included in the multivariate logistic regression to assess independent risk factors. The receiver operating characteristic (ROC) curve was generated to calculate the sensitivity and specificity and the area under the ROC curve (AUC) and determine the cut-off points for dividing. Subsequently, CRP was transformed into a binary categorical variable based on the ROC analysis’s threshold.

To adjust for the differences in baseline characteristics and to minimize bias, propensity score matching (PSM) analysis was implemented using a ratio of 1:1 nearest-neighbor matching, with a match tolerance of 0.01 [35]. We included the following variables in the PSM: age, sex, smoking, diabetes, coronary heart disease (CHD), prior anticoagulation or antiplatelet therapy, time from symptom onset to initial CT, baseline Glasgow coma (GCS) score, baseline ICH volume, ICH location, Hb, PLT, PT, INR, aPTT, d-dimer, CRP, hematoma expansion, treatment, hemiplegia, prophylactic use of low-dose subcutaneous heparin. Then, weighted multivariate logistic regression models and linear analysis determined correlations between CRP and LEDVT. Model 1 was unadjusted. Model 2 adjusted for age, sex, hypertension, smoking, diabetes, and CHD. Model 3 adjusted for variables with a *P*-value < 0.10 in univariate analysis. Model 4 was the full model that adjusted for age, sex, smoking, diabetes, coronary heart disease, prior anticoagulation or antiplatelet therapy, time from symptom onset to initial CT, baseline GCS score, baseline ICH volume, ICH location, admission laboratory tests, hematoma expansion, treatment, hemiplegia, prophylactic use of low-dose subcutaneous heparin.

The restricted cubic spline (RCS) model is a statistical analysis method utilized to generate a smooth curve illustrating the nonlinear relationship between CRP and LEDVT. The RCS model was employed in this study to analyze nonlinear trends or dose–response relationships, with the 5th, 50th, and 95th CRP identified as the three knots. Our RCS model construction was based on weighted multivariate linear regression models.

Additionally, sensitivity analyses were carried out. We performed subgroup analyses for the incidence of LEDVT, grouped by sex, age (< 60 yrs. or ≥ 60 yrs.), hypertension (yes or no), smoking status, and time from ICH onset to initial CT, and tested for interactions between grouping variables and CRP using likelihood ratio tests.

## Results

### Before PSM

Of the 538 patients (mean age 59.67 ± 10.64, 63.8% female) with primary ICH who met the inclusion criteria, 76 (14.13%) experienced LEDVT: 66 distal LEDVTs (86.84%), seven proximal LEDVTs (9.21%), and three mixed LEDVTs (3.95%). Five patients in the LEDVT group suffered from a PE.

Univariate and multivariate analyses were undertaken to determine the independent predictive of LEDVT in patients with primary ICH (Additional file 1: Table S1). The demographic and clinical characteristics of the non-LEDVT and LEDVT groups were compared and summarized in Additional file 1: Table S1. In the multilinearity diagnosis, the highest VIF (2.84) and lowest tolerance (0.35) occurred in the baseline ICH volume. No strong multilinearity was found in the variables (Additional file 2: Table S2). The multivariate analysis results were adjusted for the odds ratio (OR) of variables with* P* < 0.10 in the univariate analysis, including age, smoking, time from ICH onset to initial CT, baseline GCS score, baseline ICH volume, platelet (PLT)**,** INR, aPTT, d-dimer, CRP, hemiplegia, and treatments. After adjustment in the multivariate model (Table 1), CRP [OR 1.116, 95% confidence interval (CI) 1.054–1.181, *P* < 0.001] was statistically associated with LEDVT (Additional file 1: Table S1).

**Table 1:** Baseline characteristics of patients with ICH based on CRP levels after propensity score matching

Characteristics	Before propensity score matching			After propensity score matching		
	CRP < 1.59 mg/L	CRP ≥ 1.59 mg/L	*P*-value	CRP < 1.59 mg/L	CRP ≥ 1.59 mg/L	*P*-value
N	306	232		195	195	
Age(yrs), mean ± SD	59.0 ± 10.3	60.6 ± 11.0	0.08	59.5 ± 10.6	60.0 ± 10.8	0.664
Sex (*N*, %)			0.374			0.835
Male	200 (65.4)	143 (61.6)		119 (61.0)	121 (62.1)	
Female	106 (34.6)	89 (38.4)		76 (39.0)	74 (37.9)	
Hypertension (*N*, %)			0.857			1.0
No	98 (32.0)	76 (32.8)		62 (31.8)	62 (31.8)	
Yes	208 (68.0)	156 (67.2)		133 (68.2)	133 (68.2)	
Diabetes (*N*, %)			0.714			0.667
No	259 (84.6)	199 (85.8)		168 (86.2)	165 (84.6)	
Yes	47 (15.4)	33 (14.2)		27 (13.8)	30 (15.4)	
CHD (*N*, %)			0.98			0.804
No	294 (96.1)	223 (96.1)		187 (95.9)	186 (95.4)	
Yes	12 (3.9)	9 (3.9)		8 (4.1)	9 (4.6)	
Smoking (*N*, %)			0.132			0.551
No	272 (88.9)	196 (84.5)		171 (87.7)	167 (85.6)	
Yes	34 (11.1)	36 (15.5)		24 (12.3)	28 (14.4)	
Alcohol (*N*, %)			0.895			0.859
No	278 (90.8)	210 (90.5)		178 (91.3)	177 (90.8)	
Yes	28 (9.2)	22 (9.5)		17 (8.7)	18 (9.2)	
Prior anticoagulation or antiplatelet therapy (*N*, %)			0.121			0.535
No	291 (95.1)	213 (91.8)		184 (94.4)	181 (92.8)	
Yes	15 (4.9)	19 (8.2)		11 (5.6)	14 (7.2)	
Time from symptom onset to initial CT, hours, median (IQR)	4.0 (3.0–5.0)	4.0 (3.0–6.0)	0.007	4.0 (3.0–5.5)	4.0 (3.0–5.0)	0.724
Baseline GCS score, median (IQR)	11.0 (8.0–13.0)	10.0 (8.0–13.0)	0.345	11.0 (8.0–13.0)	10.0 (8.0–13.0)	0.573
Baseline ICH volume, mL, median (IQR)	14.2 (6.0–30.2)	17.1 (7.1–37.8)	0.1	14.3 (5.8–33.7)	16.1 (6.4–35.2)	0.571
Intraventricular hemorrhage (*N*, %)			0.844			1.0
No	220 (71.9)	165 (71.1)		139 (71.3%)	139 (71.3%)	
Yes	86 (28.1)	67 (28.9)		56 (28.7%)	56 (28.7%)	
ICH location (*N*, %)			0.406			0.886
Lobar	41 (13.4)	37 (15.9)		28 (14.4)	29 (14.9)	
Deep	265 (86.6)	195 (84.1)		167 (85.6)	166 (85.1)	
Admission Laboratory						
Hemoglobin, g/L, mean ± SD	154.1 ± 23.4	150.7 ± 22.8	0.099	150.8 ± 22.6	151.8 ± 21.7	0.679
Platelet,10^9^/L, mean ± SD	175.5 ± 56.7	176.4 ± 69.9	0.864	173.6 ± 56.0	176.4 ± 68.4	0.659
Prothrombin time, seconds, mean ± SD	11.7 ± 2.9	11.8 ± 2.5	0.795	11.7 ± 2.7	11.8 ± 2.7	0.792
INR (IQR)	1.3 ± 4.7	1.1 ± 0.2	0.414	1.0 ± 0.3	1.0 ± 0.3	0.685
aPTT, seconds, mean ± SD	24.7 ± 5.2	26.3 ± 5.2	< 0.001	25.5 ± 5.1	25.7 ± 5.1	0.737
d-dimer, ug/mL, median (IQR)	0.9 ± 2.3	2.0 ± 6.5	0.006	1.1 ± 2.8	1.1 ± 1.5	0.952
CRP, median (IQR), mg/L	0.7 ± 0.4	8.1 ± 14.9	< 0.001	0.9 ± 0.4	6.0 ± 5.3	< 0.001
Hematoma expansion (*N*, %)			0.26			0.739
No	269 (87.9)	211 (90.9)		176 (90.3)	174 (89.2)	
Yes	37 (12.1)	21 (9.1)		19 (9.7)	21 (10.8)	
Treatment (*N*, %)			0.527			0.839
Conservative therapy (*N*, %)	168 (54.9)	121 (52.2)		107 (54.9)	105 (53.8)	
Surgery (*N*, %)	138 (45.1)	111 (47.8)		88 (45.1)	90 (46.2)	
Hemiplegia (*N*, %)			0.508			0.838
No	138 (45.1)	98 (42.2)		86 (44.1)	84 (43.1)	
Yes	168 (54.9)	134 (57.8)		109 (55.9)	111 (56.9)	
Prophylactic use of low-dose subcutaneous heparin, (*N*, %)			0.712			0.798
No	240 (78.4)	185 (79.7)		158 (81.0)	156 (80.0)	
Yes	66 (21.6)	47 (20.3)		37 (19.0)	39 (20.0)	
LEDVT (*N*, %)			< 0.001			< 0.001
No	298 (97.4)	164 (70.7)		187 (95.9)	147 (75.4)	
Yes	8 (2.6)	68 (29.3)		8 (4.1)	48 (24.6)	

*aPTT* activated partial thromboplastin time, *CHD* coronary heart disease, *CT* computed tomography, *CRP* C-reactive protein, *GCS score* Glasgow coma score, *INR* international normalized ratio, *LEDVT* lower-extremity deep venous thrombosis, *ICH* intracerebral hemorrhage, *SD* standard deviations, *IQR* interquartile range

**Fig. 2:**
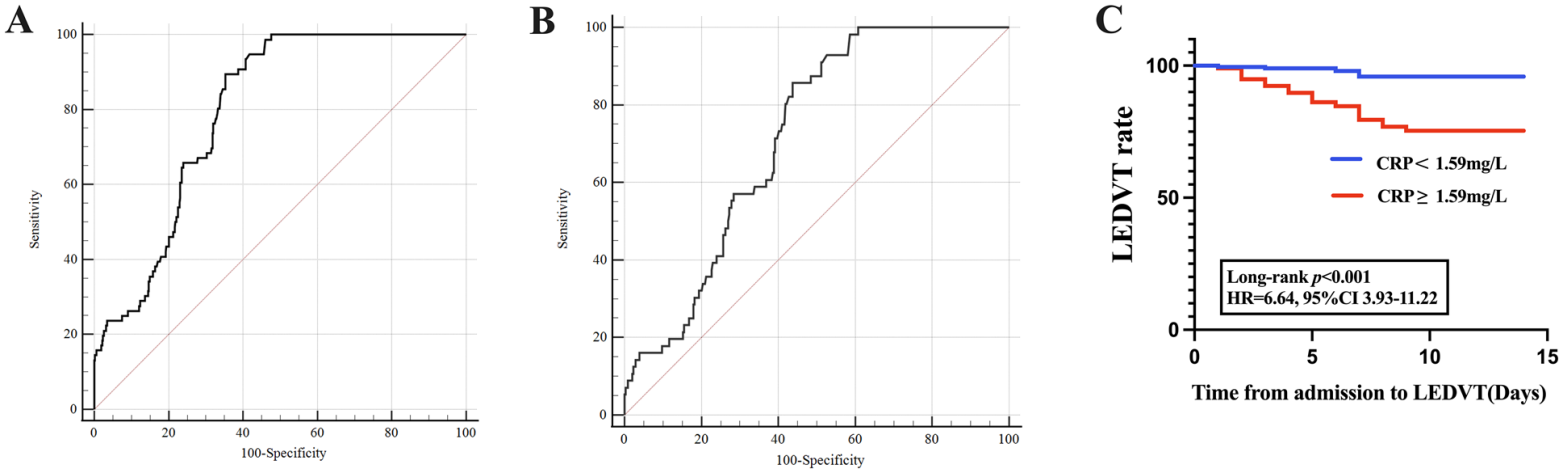
Association of peripheral blood C-reactive protein with LEDVT. **A** The optimal cut-off value of CRP calculated by the ROC curve to predict LEDVT was 1.59 mg/L (AUC: 0.795, 95%CI 0.759–0.829, *P* < 0.001; the sensitivity was 89.47%, and the specificity was 64.72%) in patients with primary ICH before propensity score matching. **B** After propensity score matching, the ROC curve revealed an AUC of 0.717 (95% CI 0.669–0.761, *P* < 0.001) for CRP to predict LEDVT with a sensitivity of 85.71% and specificity of 56.29%. **C**. Cumulative LEDVT rates within 14 days of ICH admission based on CRP thresholds using Kaplan–Meier analysis (Hazard ratio = 6.64, 95% CI 3.93–11.22, Long-rank *P* <0.001) after propensity score matching. *AUC* the area under the curve, *CI* confidence interval, *CRP* C-reactive protein, *LEDVT* lower-extremity deep venous thrombosis, *OR* odds ratio, *ROC* receiver operating characteristic

A ROC curve was generated to evaluate the predictive power of CRP further. The ROC curve calculated the optimal threshold value of CRP for predicting LEDVT at 1.59 mg/L (AUC: 0.795, 95%CI 0.759–0.829, *P* < 0.001; the sensitivity was 89.47%, and the specificity was 64.72%; Fig. 2A) in ICH patients. We used the threshold value of CRP (1.59 mg/L) to subdivide the patients into two groups: (1) patients with CRP < 1.59 mg/L, and (2) patients with CRP ≥ 1.59 mg/L. Before PSM, patients with CRP ≥ 1.59 mg/L showed a higher prevalence of LEDVT and old age than patients with CRP < 1.59 mg/L. If a variable inflation factor (VIF) is greater than 5 or the tolerance is less than 0.2, it suggests the presence of multiple collinearities.

### After PSM

We conducted a one-to-one PSM analysis to reduce the effect of additional confounding factors on the relationship between CRP and LEDVT. After the PSM, 195 patients were enrolled in each group. Table 1 illustrates the comparison of baseline characteristics before and after PSM. No significant differences were found for all the variables (Table 1). Overall were excluded 36.3% of patients with CRP < 1.59 mg/L and 15.9% of patients with CRP ≥ 1.59 mg/L. After PSM, in the comparisons between patients with CRP ≥ 1.59 mg/L and those with CRP < 1.59 mg/L, the LEDVT events within 14 days after the acute ICH episode were 48 (24.6%) and 8 (4.1%), respectively (Table 1). The ROC curve revealed an AUC of 0.717 (95% CI 0.669–0.761, *P* < 0.001; Fig. 2B) for CRP to predict LEDVT with a sensitivity of 85.71% and specificity of 56.29%. Cumulative LEDVT rates within 14 days of ICH admission based on CRP thresholds [hazard ratio (HR) = 6.64, 95%CI 3.93–11.22, long-rank *P* < 0.001] using Kaplan–Meier analysis are presented in Fig. 2C.

### Weighted univariate logistics analysis of LEDVT‐related variable after PSM

Weighted univariate logistic regression analysis was conducted to observe the associations between CRP and LEDVT. Age was associated with LEDVT, with an OR of 1.0 (95% CI 1.00–1.1, *P* < 0.001, Table 2). Patients who had a history of smoking and CRP ≥ 1.59 mg/L were more likely to develop LEDVT, with ORs of 2.6 (95% CI 1.3–5.2, *P* = 0.007), 7.6 (95%CI 3.5–16.6,* P* < 0.001), respectively (Table 2).

**Table 2:** Weighted univariate logistics analysis of LEDV-related variable

Characteristics	OR(95%CI)	*P*-value
Age	1.0 (1.0–1.1)	0.001
Sex		
Male	Reference	
Female	1.1 (0.6–2.0)	0.665
Hypertension		
No	Reference	
Yes	1.1 (0.6–2.0)	0.803
Diabetes		
No	Reference	
Yes	0.8 (0.3–1.9)	0.629
CHD		
No	Reference	
Yes	0.4 (0.0–2.8)	0.328
Smoking		
No	Reference	
Yes	2.6 (1.3–5.2)	0.007
Alcohol		
No	Reference	
Yes	1.6 (0.6–3.8)	0.322
Prior anticoagulation or antiplatelet therapy		
No	Reference	
Yes	1.5 (0.6–4.3)	0.409
Time from symptom onset to initial CT	1.1 (1.0–1.2)	0.007
Baseline GCS score	0.9 (0.8–1.0)	0.056
Baseline ICH volume	1.0 (1.0, 1.0)	0.387
Intraventricular hemorrhage		
No	Reference	
Yes	1.3 (0.7–2.4)	0.353
ICH location		
Lobar	Reference	
Deep	1.2 (0.5–2.9)	0.629
Admission laboratory		
Hemoglobin	1.0 (1.0–1.0)	0.645
Platelet	1.0 (1.0–1.0)	0.697
Prothrombin time	1.0 (0.9–1.1)	0.713
INR	0.9 (0.3–3.0)	0.854
aPTT	1.0 (0.9–1.0)	0.722
d-dimer	1.1 (0.9–1.2)	0.349
C-reactive protein		
< 1.59 mg/L	Reference	
≥ 1.59 mg/L	7.6 (3.5–16.6)	< 0.001
Hematoma expansion		
No	Reference	
Yes	0.6 (0.2–1.9)	0.41
Treatment		
Conservative therapy	Reference	
Surgery	1.5 (0.8–2.6)	0.2
Hemiplegia		
No	Reference	
Yes	1.5 (0.8–2.6)	0.2
Prophylactic use of low-dose subcutaneous heparin		
No	Reference	
Yes	0.9 (0.4–1.8)	0.739

*aPTT* activated partial thromboplastin time, *CHD* coronary heart disease, *CT* computed tomography, *CRP* C-reactive protein, *GCS score* Glasgow coma score, *INR* international normalized ratio, *LEDVT* lower-extremity deep venous thrombosis, *ICH* intracerebral hemorrhage, *SD* standard deviations, *IQR* interquartile range

### Weighted multivariable logistics regression analysis of the association between CRP and LEDVT

Table 3 displays the findings of the weighted multivariable logistic regression models of the incidence of LEDVT and CRP. A significant positive correlation was found between the incidence of LEDVT and CRP in four models. Higher CRP levels (≥ 1.59 mg/L) have an increased risk of LEDVT than those patients with CRP < 1.59 mg/L, with ORs and CIs of 7.6 (3.5–16.6) for model 1, 8.0 (3.6–17.7) for model 2, and 8.5 (3.8–19.1) for model 3, 10.8 (4.5–25.8) for model 4, respectively (Table 3). Consequently, the results of the regression models indicate that higher CRP levels (≥ 1.59 mg/L) are correlated with an increased likelihood of experiencing LEDVT.

**Table 3:** ORs (95% CIs) of the association between CRP and LEDVT in four models

	Model1		Model 2		Model 3		Model 4	
	OR (95%CI)	*P* value	OR (95%CI)	*P* value	OR (95%CI)	*P* value	OR (95%CI)	*P* value
< 1.59 mg/L	Reference		Reference		Reference		Reference	
≥ 1.59 mg/L	7.6 (3.5–16.6)	< 0.001	8.0 (3.6–17.7)	< 0.001	8.5 (3.8–19.1)	< 0.001	10.8 (4.5–25.8)	< 0.001

Four logistic regression models were utilized for analysis: model 1 with no covariate adjustment. Model 2 adjusted for age, sex, hypertension, smoking, diabetes, and CHD. Model 3 adjusted for variables with a *P*-value < 0.10 in univariate analysis, including age, baseline GCS score, time from onset to initial CT, and smoking. Model 4 was the full model that adjusted for age, sex, smoking, diabetes, CHD, prior anticoagulation or antiplatelet therapy, time from symptom onset to initial CT, baseline GCS score, baseline ICH volume, ICH location, Hb, PLT, PT, INR, aPTT, d-dimer and CRP), hematoma expansion, treatment, hemiplegia, prophylactic use of low-dose subcutaneous heparin

*CI* confidence interval, *OR* odds ratio

### Dose–response relationship between CRP and the risk of LEDVT

**Fig. 3:**
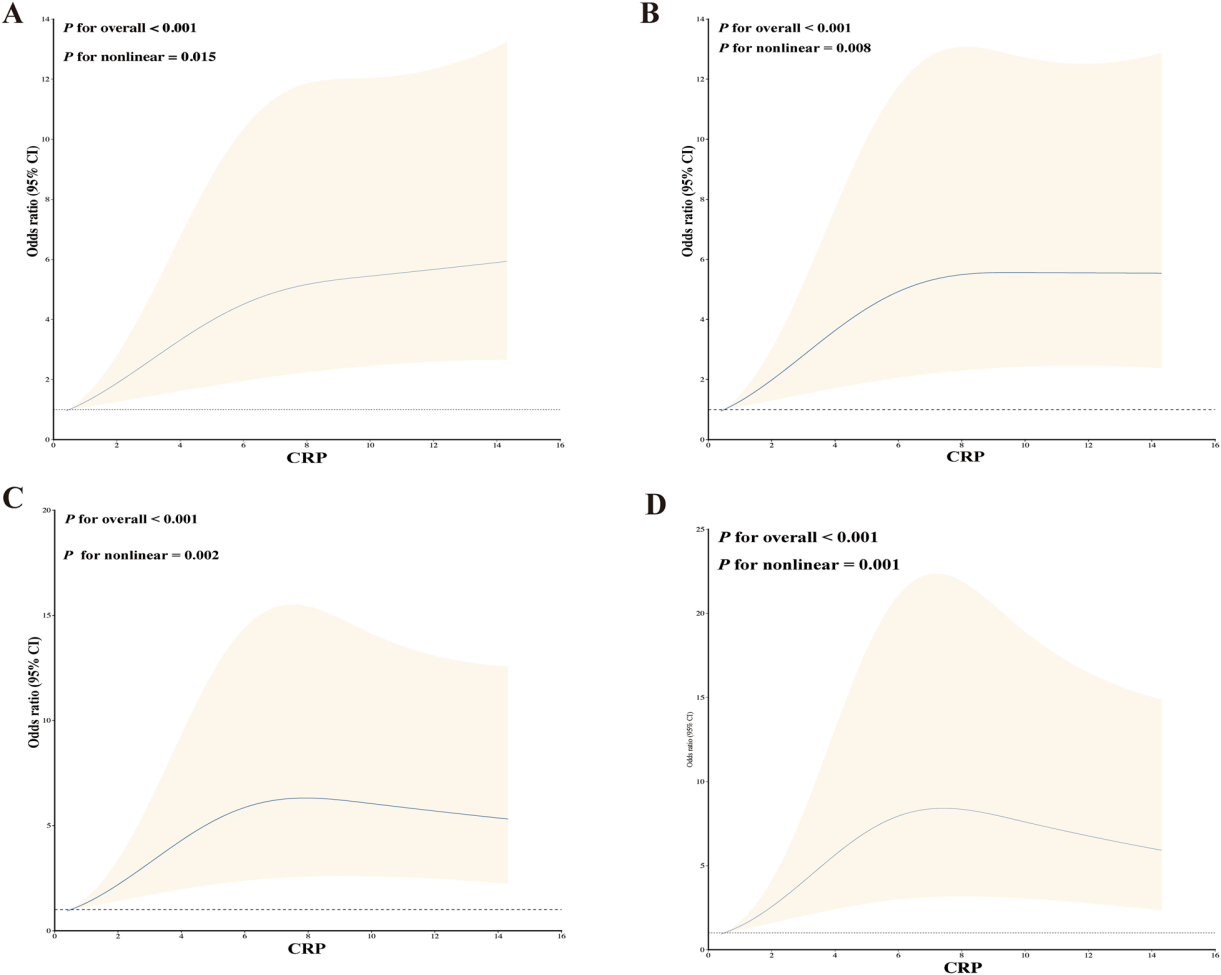
Restricted cubic spline modeling of the relationship between CRP and LEDVT. A Model 1 for LEDVT; B Model 2 for LEDVT; C Model 3 for LEDVT; D Model 4 for LEDVT. Model 1 was unadjusted. Model 2 was adjusted for age, sex, hypertension, smoking, diabetes, and coronary heart disease. Model 3 was adjusted for variables with a *P*-value < 0.10 in univariate analysis, including age, GCS score, time from onset to initial CT, and smoking. Model 4 was the full model that adjusted for age, sex, smoking, diabetes, CHD, prior anticoagulation or antiplatelet therapy, time from symptom onset to initial CT, baseline GCS score, baseline volume, ICH location, Hb, PLT, PT, INR, aPTT, d-dimer and CRP), hematoma expansion, treatment, hemiplegia, prophylactic use of low-dose subcutaneous heparin

Our study employed RCS plots to show the associations between CRP and LEDVT. Four models were developed to adjust for confounding factors based on weighted multivariable logistic regression models. Results from all four nonlinear RCS models demonstrated a significant nonlinear association between CRP levels and an increased risk of LEDVT (model 1, *P* for overall < 0.001, *P* for nonlinear = 0.015; model 2, *P* for overall < 0.001, *P* for nonlinear = 0.008; model 3, *P* for overall < 0.001, *P* for nonlinear = 0.002; model 4, *P* for overall < 0.001, *P* for nonlinear = 0.001, Fig. 3A–D). The increased risk of LEDVT was associated with elevated CRP levels.

### Subgroup analysis of the association between CRP and LEDVT

**Fig. 4:**
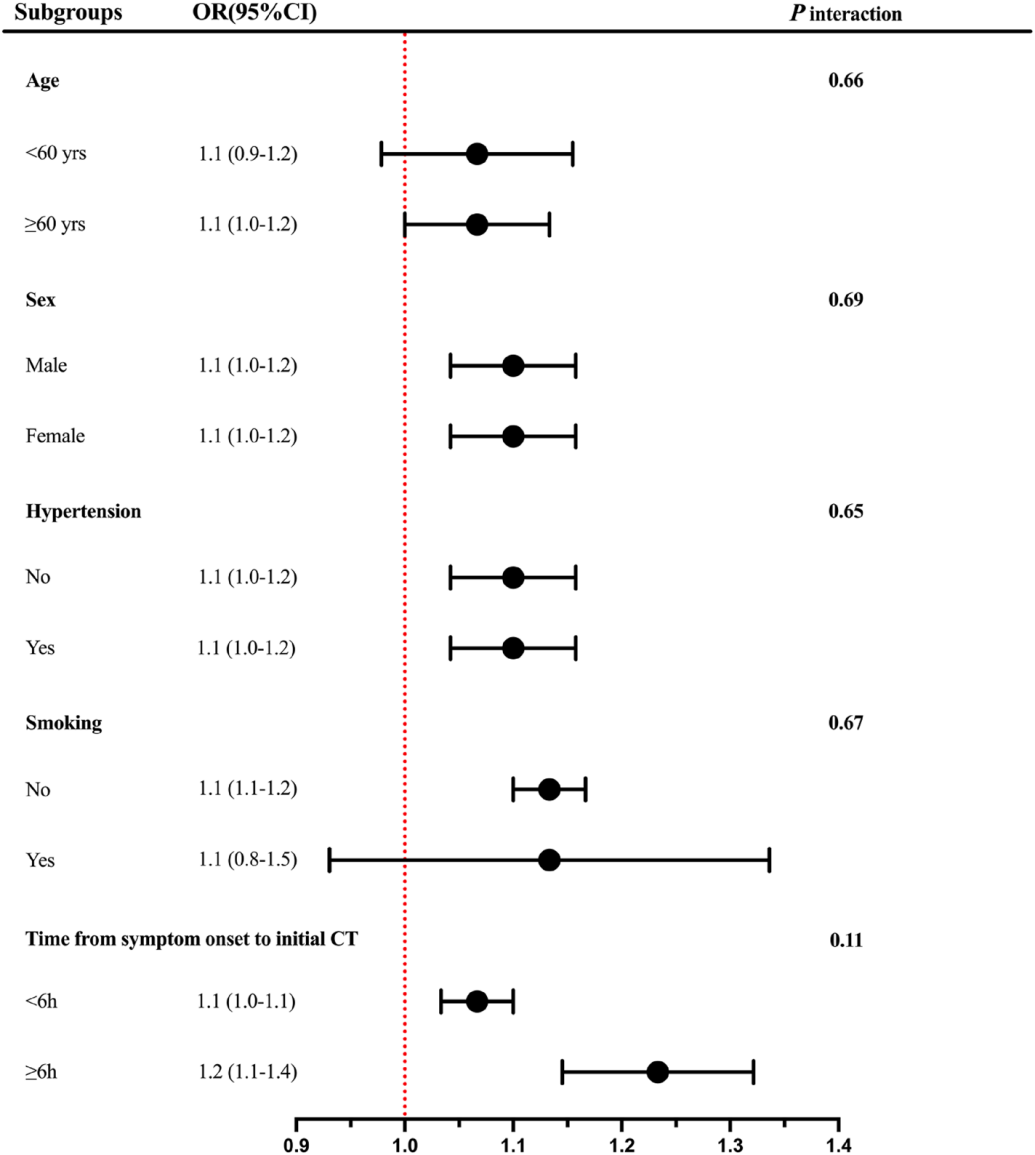
Subgroup analysis for the relationship between CRP and LEDVT. Subgroup analysis was based on Model 4 that adjusted for age, sex, smoking, diabetes, coronary heart disease, prior anticoagulation or antiplatelet therapy, time from symptom onset to initial CT, baseline GCS score, baseline ICH volume, ICH location, Hb, PLT, PT, INR, aPTT, d-dimer, CRP, hematoma expansion, treatment, hemiplegia, prophylactic use of low-dose subcutaneous heparin, and CRP, C-reactive protein except the stratification factor itself

Subgroup analyses were undertaken to explore the study results further and identify any special subgroups in our study. As shown in Fig. 4, our findings indicated no significant associations were observed between CRP and LEDVT when stratifying by sex (*P* = 0.69), age (*P* = 0.66), hypertension (*P* = 0.65), smoking status (*P* = 0.67), and time from symptom onset to initial CT (*P* = 0.11). The results indicated a consistent positive link between CRP and LEDVT events in patients with diverse demographics and disease statuses.

## Discussion

In the current study, we used PSM analysis, weighted logistic regression analyses, and the RCS model to assess the association between admission CRP levels and LEDVT in patients with primary ICH. Our results demonstrated that admission CRP level was associated with an increased risk of LEDVT after PSM. The ROC curve revealed an AUC of 0.717 for CRP to predict LEDVT with a sensitivity of 85.71% and specificity of 56.29%. After adjusting for all confounding variables, the occurrence of LEDVT in patients with ICH with higher CRP levels (≥ 1.59 mg/L) was 10.8 times higher than those with lower CRP levels. Furthermore, higher CRP levels (CRP ≥ 1.59 mg/L) indicated a stable positive association with LEDVT events in the multivariate logistic regression analysis, weighted generalized additive model, and RCS models. Interaction tests revealed no statistically significant effects of demographics and disease status on the association between CRP and LEDVT events.

Numerous population-based prospective cohort studies have previously documented the relationships between circulating levels of CRP and the risk of subsequent VTE, but the results have been contradictory [7, 36]. Folsom A. R. and colleagues demonstrated that CRP was associated with total VTE in multivariable analyses in the Atherosclerosis Risk in Communities (ARIC) cohort [36]. Elevated circulating CRP was associated with an increased risk of VTE in a prospective study and meta-analysis , and agreed with a linear dose–response relationship [7].

Interestingly, a review found weak evidence for a causal role of CRP in VTE etiology [21]. The Tromsø study indicated no association between serum CRP levels and subsequent risk of VTE [22]. Genetic polymorphisms that increase CRP levels have not been associated with increased VTE risk [23]. However, we found that the higher initial CRP levels (CRP ≥ 1.59 mg/L) in ICH patients were associated with an increased risk of LEDVT, and a significant nonlinear association between CRP levels and the risk of LEDVT was identified. Possible reasons for the inconsistent findings across previous studies and ours could be explained by several aspects. First, the two studies are not on the same subject. We studied ICH patients in a stressful state, whereas they were based on the general population. Secondly, the composition of the population studied was different. Third, the primary outcome of our study was LEDVT, whereas their study was based on VTE. LEDVT is just one of the most prevalent types of VTE. Fourth, the follow-up periods in the different studies are not the same. Our assessment of LEDVT was that it occurred within 14 days of onset, whereas they had a variety of different and inconsistent follow-up periods for outcomes (TVE). Based on the nature of the retrospective study, we cannot conclude that elevated CRP may be causally related to LEDVT but may be a risk biomarker of LEDVT.

Hypercoagulation, stasis, and endothelial dysfunction (Virchow’s triad) are the primary drivers of DVT [37]. Thrombosis is a sophisticated procedure that predominantly involves coagulation and inflammation. Inflammation potentially affects all stages of the coagulation pathway. After acute inflammation, the coagulation cascade is triggered, contributing to thrombotic events [4]. Thrombosis is exacerbated by inflammatory aggregation [37].

An acute ICH can activate the systemic inflammatory response [38]. During the immediate aftermath of an acute ICH episode, it induces a systemic inflammatory response, leading to increased peripheral blood inflammatory indicators. On the other hand, the ICH hematoma component triggers an inflammatory response in the surrounding brain tissue, which together with the ICH hematoma, exacerbates the brain tissue damage and further increases the inflammatory response in the peripheral blood [27]. Inflammatory responses increase coagulation activation among ICH patients. Inflammation on the blood vessel wall triggers thrombus formation in the vein [39]. Venous wall inflammation is the probable initial cause of VTE formation [40]. Our previous study has identified leukocytes upon admission as an independent predictor of risk for LEDVT in elderly patients with primary ICH [4], supporting that inflammation significantly contributes to the occurrence of LEDVT [2, 13]. Previous studies have identified the association between VTE and inflammation markers such as CRP, Interleukin 6 (IL-6), IL-8, tumor necrosis factor-α (TNF-α), and Neutrophil extracellular traps (NETs) [41–43].

CRP is an anti-inflammatory, acute-phase protein synthesized by hepatocytes and regarded as a proven clinical marker of systemic inflammation [23]. CRP is generated in the liver induced by IL-6, IL-8, and TNF-α. CRP is a downstream biomarker of elevated IL-1, IL-6, and TNF-α [41, 42]. It increases 10,000-fold in 6 h (h), with a half-life of 19 h, and decomposes at a rate independent of blood concentration [41, 42]. First, inflammation is well known to play a pivotal role in all stages of DVT formation. CRP actively participates by triggering the complement system, inducing apoptosis, vascular cell activation, leukocyte recruitment, and platelet activation aggregation, ultimately leading to thrombosis [42]. Elevated CRP signifies an orchestrated inflammatory response with the potential to upregulate proinflammatory cytokines, notably IL-6. The initiation of the inflammatory cascade may, in turn, precipitate endothelial dysfunction and activate coagulation pathways, thereby fostering a prothrombotic microenvironment.

Additionally, under the stressful condition of ICH, CRP induces tissue factor synthesis and expression in human peripheral blood monocytes, thereby promoting the formation of LEDVT [22]. Second, CRP impairs endothelial-dependent vascular relaxation by inhibiting nitric oxide and increasing endothelin-1 production, which leads to endothelial dysfunction and consequent LEDVT [41, 44]. CRP has been implicated in endothelial dysfunction, a phenomenon characterized by impaired anticoagulant properties of the vascular endothelium. This impairment may enhance platelet adhesion and aggregation, thereby facilitating thrombus formation in the deep veins of the lower extremities [45]. Third, higher CRP levels are a secondary acute-phase response to LEDVT formation and induce tissue factor expression by monocytes, smooth muscle cells, and endothelial cells [7]. CRP levels are positively linked to thrombus incidence, extent, and volume in cancer patients [19, 46]. Several studies have illustrated that elevated CRP levels correlate remarkably well with extensive incidences of VTE, including LEDVT [7, 20, 36, 41]. Immobilization, surgery, and hypercoagulation disorders after ICH develop and aggravate LEDVT. Patients with primary ICH often present with underlying comorbidities, such as hypertension and diabetes, independently associated with increased CRP levels and heightened thrombotic risk. The complex interplay between CRP, comorbidities, and the resultant hypercoagulable state may synergistically elevate the risk of lower-extremity DVT.

Furthermore, elevated CRP levels have been associated with impaired fibrinolysis, the physiological process responsible for clot dissolution. This inhibition of fibrinolysis may contribute to the persistence and growth of thrombi in the lower extremities, amplifying the risk of LEDVT [47]. CRP can directly or indirectly activate platelets, pivotal players in thrombus formation. Activated platelets contribute significantly to the intricate process of thrombus development, potentially intensifying the risk of DVT [48]. Last, serum CRP was positively correlated with NETs levels [49, 50]. NETs released from activated neutrophils have been documented to promote immune thrombosis by interacting with macrophages, platelets, and vascular endothelial cells as a pathogenetic mechanism to induce VTE [43, 51]. On the other hand, NETs contain myeloperoxidases, enzymes, and histones, which have bactericidal, procoagulant, proinflammatory and antifibrinolytic effects that help to stabilize clot production [43, 51]. Taken together, the association between heightened CRP levels on admission and an augmented risk of lower-extremity DVT in patients with primary intracerebral hemorrhage involves a myriad of interconnected mechanisms. These encompass inflammatory cascades, endothelial dysfunction, impaired fibrinolysis, platelet activation, and the influence of comorbidities. A more nuanced comprehension of these intricate processes may pave the way for targeted therapeutic interventions to mitigate the elevated risk and improve overall clinical outcomes.

### Strengths and limitations

The study has several strengths and limitations to consider when weighing the implications of the results. First, this study is a two-center, large-sample cohort study based on patients with ICH, with substantial outcomes and longitudinal follow-up. Second, this study is a large population with comprehensive demographic characteristics and cardiopulmonary, radiological, and laboratory chemistry data documentation. Despite the strengths of this study, some limitations exist, including those inherent to a retrospective observational study design. Firstly, due to its retrospective observational analysis, we could not address causal associations or rule out the presence of unmeasured confounding factors. Secondly, this study only examined the relationship between CRP levels upon admission and the subsequent occurrence of LEDVT rather than the dynamic changes in CRP concerning LEDVT. Thirdly, some ICH patients with LEDVT had admission CRP levels (reference range: 0–10 mg/L) within the normal range, which makes the results difficult to interpret directly. More profound mechanisms deserve further study. Fourthly, although venography is still considered the gold standard for diagnosing DVT or LEDVT, it is invasive and is not a cost-effective method of routine assessment. Due to the severity of ICH and the possible use of heparin during venography, we do not routinely perform venography, preferring to employ CDU to diagnose LEDVT. Finally, this model of the association between low-grade systemic inflammation, as measured by serum CRP, and the risk of LEDVT in patients with primary ICH was performed in the Chinese population that characteristically have fewer low-grade proinflammatory factors like diabetes, smoking, etc., except for hypertension and that its use should be revalidated in other populations.

## Conclusions

The higher initial CRP levels in patients with primary ICH are significantly associated with an increased risk of LEDVT and may help identify high-risk patients with LEDVT. Clinicians should be vigilant to enable early and effective intervention in patients at high risk of LEDVT. However, further large-scale, or randomized studies are warranted to validate these findings and to determine whether changes in CRP levels over time are related to the occurrence and progression of LEDVT. Furthermore, the inspiration gained from this study makes it very imperative to frequently monitor blood markers and perform ultrasonography to facilitate early diagnosis and active prevention of LEDVT. In future studies, we expect to investigate whether elevated CRP levels directly contribute to LEDVT or whether CRP is involved in the progression of LEDVT. In addition, we need to further analyze any changes in CRP levels after treatment for LEDVT.

### Supplementary Information

**Additional file 1: Table S1.** Univariate and multivariate analyses of association with LEDVT in patients with primary intracerebral hemorrhage.

**Additional file 2: Table S2.** Multicollinearity test for the factors of a multivariate model.

## Data Availability

The datasets generated and/or analyzed during the current study are not publicly available due to privacy or ethical restrictions, but are available from the corresponding author on reasonable request.
