# Supplementary material for: C-reactive protein is a predictor for lower-extremity deep venous thrombosis in patients with primary intracerebral hemorrhage
Source: Eur J Med Res. 2024 Jun 6;29:311. doi: 10.1186/s40001-024-01842-3 (PMC11157878; doi:10.1186/s40001-024-01842-3)
Supplement: Supplementary file 1 — Additional file 1: Table S1. Univariate and multivariate analyses of association with LEDVT in patients with primary intracerebral hemorrhage. [file 40001_2024_1842_MOESM1_ESM.doc]

**TABLE S1 ǀ Univariate and multivariate analyses of association with LEDVT in patients with primary intracerebral hemorrhage**

|  | **Univariate analysis** | | | **Multivariate analysis ^a^** | | |
| --- | --- | --- | --- | --- | --- | --- |
| **Characteristics** | **Non-LEDVT**  **(N=462)** | **LEDVT**  **(N=76)** | ***P* value** | **Odds ratio** | **95%Confidence interval** | ***P* value** |
| **Age(yrs)**, **mean±SD** | 58.84±10.60 | 64.72±9.45 | <0.001 | 1.055 | 1.025-1.086 | <0.001**^*^** |
| **Sex (N, %)** |  |  | 0.528 |  |  |  |
| **Male** | 297(64.3) | 46(60.5) |  |  |  |  |
| **Female** | 165(35.7) | 30(39.5) |  |  |  |  |
| **Medical history** |  |  |  |  |  |  |
| **Hypertension (N, %)** | 221(67.5) | 52(68.4) | 0.878 |  |  |  |
| **Diabetes (N, %)** | 71(15.4) | 9(11.8) | 0.423 |  |  |  |
| **Coronary heart disease** | 20(4.3) | 1(1.3) | 0.338 |  |  |  |
| **Smoking (N, %)** | 48(10.4) | 22(28.9) | <0.001 | 0.314 | 0.155-0.632 | 0.001**^*^** |
| **Alcohol (N, %)** | 41(8.9) | 9(11.8) | 0.409 |  |  |  |
| **Prior anticoagulation or antiplatelet therapy (N, %)** | 26(5.6) | 8(10.5) | 0.104 |  |  |  |
| **Admission vital signs** |  |  |  |  |  |  |
| **Time from symptom onset to initial CT, hours, median (IQR)** | 4.0(3.0-5.0) | 5.0(4.0-8.0) | <0.001 | 1.105 | 1.034-1.180 | 0.003**^*^** |
| **Baseline** **Glasgow coma score, median (IQR)** | 11.0(8.0-13.0) | 9.0(8.0-11.75) | 0.003 | 0.953 | 0.840-1.080 | 0.451 |
| **Baseline ICH volume, ml, median (IQR)** | 15.02(5.96-30.57) | 27.05(10.06-41.68) | 0.002 | 0.997 | 0.981-1.014 | 0.746 |
| **ICH location (N, %)** |  | 0.255 | 0.141 |  |  |  |
| **Deep** | 393(85.1) | 67(88.2) |  |  |  |  |
| **Lobar** | 69(14.9) | 9(11.8) |  |  |  |  |
| **Admission Laboratory** |  |  |  |  |  |  |
| **Hemoglobin, g/L, mean±SD** | 152.73±22.55 | 151.95±27.05 | 0.786 |  |  |  |
| **Platelet,10^9^/L, mean±SD** | 177.86±62.76 | 163.72±60.87 | 0.068 | 0.998 | 0.993-1.002 | 0.347 |
| **Prothrombin time, seconds, mean±SD** | 11.76±2.89 | 11.72±1.11 | 0.909 |  |  |  |
| **International normalized ratio (IQR)** | 1.02(0.95-1.07) | 1.04(0.98-1.12) | 0.009 | 0.416 | 0.091-1.910 | 0.259 |
| **Activated partial thromboplastin time, seconds, mean±SD** | 25.16±5.32 | 26.54±4.68 | 0.033 | 1.031 | 0.971-1.094 | 0.316 |
| **D-dimer, ug/ml, median (IQR)** | 1.08±2.58 | 3.31±10.41 | <0.001 | 1.033 | 0.982-1.086 | 0.215 |
| **C-reactive protein, median (IQR), mg/L** | 0.96(0.42-2.56) | 3.31(1.89-9.34) | <0.001 | 1.116 | 1.054-1.181 | <0.001 |
| **Prophylactic use of low-dose subcutaneous heparin, (N, %)** | 100(21.6) | 13(17.1) | 0.368 |  |  |  |
| **Treatment** |  |  | 0.028 | 0.491 | 0.222-1.082 | 0.078 |
| **Conservative therapy (N, %)** | 257(55.6) | 32(42.1) |  |  |  |  |
| **Surgery (N, %)** | 205(44.4) | 44(57.9) |  |  |  |  |
| **Hemiplegia (N, %)** | 251(54.3) | 51(67.1) | 0.038 | 0.694 | 0.310-1.551 | 0.373 |
| **Hematoma expansion (N, %)** | 54(11.7) | 4(5.3) | 0.111 |  |  |  |

**^a^Adjusted covariates: age, time from symptom onset to initial CT, baseline Glasgow coma score, baseline volume, activated partial thromboplastin time, fibrinogen degradation products, D-dimer, C-reactive protein, mechanical ventilation used> 48hours, hemiplegia, treatment(surgery). ^*^*P<0.05.***

**CT, computed tomography; LEDVT, lower-extremity deep venous thrombosis; GCS, Glasgow Coma Scale; ICH, intracerebral hemorrhage; IQR, Interquartile range; SD, Standard deviation.**
