# Supplementary material for: C-reactive protein is a predictor for lower-extremity deep venous thrombosis in patients with primary intracerebral hemorrhage
Source: Eur J Med Res. 2024 Jun 6;29:311. doi: 10.1186/s40001-024-01842-3 (PMC11157878; doi:10.1186/s40001-024-01842-3)
Supplement: Supplementary file 2 — Additional file 2: Table S2. Multicollinearity test for the factors of a multivariate model. [file 40001_2024_1842_MOESM2_ESM.docx]

**TABLE S2 ǀ Multicollinearity test for the factors of a multivariate model**

| **Independent variable** | **Multicollinearity statistics** | |
| --- | --- | --- |
|  | **Tolerance** | **Variable inflation factor** |
| **Age** | 0.81 | 1.23 |
| **Sex** | 0.76 | 1.32 |
| **Hypertension** | 0.93 | 1.08 |
| **Diabetes** | 0.94 | 1.07 |
| **Coronary heart disease** | 0.86 | 1.16 |
| **Smoking** | 0.88 | 1.14 |
| **Alcohol** | 0.97 | 1.03 |
| **Prior anticoagulation or antiplatelet therapy** | 0.84 | 1.19 |
| **Time from symptom onset to initial CT** | 0.85 | 1.18 |
| **Baseline** **Glasgow coma score** | 0.49 | 2.02 |
| **Intraventricular hemorrhage** | 0.90 | 1.11 |
| **Baseline ICH volume** | 0.35 | 2.84 |
| **ICH location** | 0.77 | 1.30 |
| **Hemoglobin** | 0.78 | 1.28 |
| **Platelet** | 0.89 | 1.12 |
| **Prothrombin time** | 0.82 | 1.22 |
| **International normalized ratio** | 0.91 | 1.10 |
| **Activated partial thromboplastin time** | 0.80 | 1.24 |
| **D-dimer** | 0.88 | 1.14 |
| **C-reactive protein** | 0.81 | 1.23 |
| **Prophylactic use of low-dose subcutaneous heparin** | 0.64 | 1.57 |
| **Treatment** | 0.49 | 2.04 |
| **Hemiplegia** | 0.46 | 2.16 |
| **Hematoma expansion** | 0.91 | 1.10 |

CT, computed tomography; CRP, C-reactive protein (CRP); lower-extremity deep venous thrombosis, LEDVT; ICH, intracerebral hemorrhage;
